# Functional Connectivity Alterations Reveal Complex Mechanisms Based on Clinical and Radiological Status in Mild Relapsing Remitting Multiple Sclerosis

**DOI:** 10.3389/fneur.2018.00690

**Published:** 2018-08-20

**Authors:** Gloria Castellazzi, Laetitia Debernard, Tracy R. Melzer, John C. Dalrymple-Alford, Egidio D'Angelo, David H. Miller, Claudia A. M. Gandini Wheeler-Kingshott, Deborah F. Mason

**Affiliations:** ^1^NMR Research Unit, Department of Neuroinflammation, Queen Square MS Centre, UCL Institute of Neurology, London, United Kingdom; ^2^Department of Electrical, Computer and Biomedical Engineering, University of Pavia, Pavia, Italy; ^3^New Zealand Brain Research Institute, Christchurch, New Zealand; ^4^Department of Medicine, University of Otago, Christchurch, New Zealand; ^5^Brain Research New Zealand, Auckland, New Zealand; ^6^Department of Psychology, University of Canterbury, Christchurch, New Zealand; ^7^Department of Brain and Behavioral Sciences, University of Pavia, Pavia, Italy; ^8^Brain Connectivity Center, IRCCS Mondino Foundation, Pavia, Italy; ^9^Brain MRI 3T Center, IRCCS Mondino Foundation, Pavia, Italy; ^10^Department of Neurology, Christchurch Hospital, Christchurch, New Zealand

**Keywords:** relapsing remitting multiple sclerosis, resting state fMRI, functional connectivity, functional impairment, resting state networks

## Abstract

Resting state functional MRI (rs-fMRI) has provided important insights into functional reorganization in subjects with Multiple Sclerosis (MS) at different stage of disease. In this cross-sectional study we first assessed, by means of rs-fMRI, the impact of overall T2 lesion load (T2LL) and MS severity score (MSSS) on resting state networks (RSNs) in 62 relapsing remitting MS (RRMS) patients with mild disability (MSSS < 3). Independent Component Analysis (ICA) followed by dual regression analysis confirmed functional connectivity (FC) alterations of many RSNs in RRMS subjects compared to healthy controls. The anterior default mode network (DMNa) and the superior precuneus network (PNsup) showed the largest areas of decreased FC, while the sensory motor networks area M1 (SMNm1) and the medial visual network (MVN) showed the largest areas of increased FC. In order to better understand the nature of these alterations as well as the mechanisms of functional alterations in MS we proposed a method, based on linear regression, that takes into account FC changes and their correlation with T2LL and MSSS. Depending on the sign of the correlation between FC and T2LL, and furthermore the sign of the correlation with MSSS, we suggested the following possible underlying mechanisms to interpret altered FC: (1) FC reduction driven by MS lesions, (2) “true” functional compensatory mechanism, (3a) functional compensation attempt, (3b) “false” functional compensation, (4a) neurodegeneration, (4b) pre-symptomatic condition (damage precedes MS clinical manifestation). Our data shows areas satisfying 4 of these 6 conditions (i.e., 1,2,3b,4b), supporting the suggestion that increased FC has a complex nature that may exceed the simplistic assumption of an underlying compensatory mechanism attempting to limit the brain damage caused by MS progression. Exploring differences between RRMS subjects with short disease duration (MS_short_) and RRMS with similar disability but longer disease duration (MS_long_), we found that MS_short_ and MS_long_ were characterized by clearly distinct pattern of FC, involving predominantly sensory and cognitive networks respectively. Overall, these results suggest that the analysis of FC alterations in multiple large-scale networks in relation to radiological (T2LL) and clinical (MSSS, disease duration) status may provide new insights into the pathophysiology of relapse onset MS evolution.

## Introduction

Multiple Sclerosis (MS) is a chronic disease characterized by the presence of multifocal inflammatory demyelinated plaques distributed over space and time within the central nervous system (CNS) (1, 2). The course of the MS disease is highly varied and unpredictable. The clinical measurement of disease progression in terms of the rate at which disability accumulates in an individual is challenging. Magnetic Resonance Imaging (MRI) has contributed significantly not only to diagnosis, by depicting white matter demyelinating lesions, but also to the study of mechanisms of disease and of functional alterations.

In a 20-year follow-up MRI study of lesion load and disability, Fisniku et al. (3) showed that a concurrent change in white matter lesion load on T2-weighted scans and expanded disability status scale (EDSS) scores in the first 5 years of the disease is indicative of long-term disability. Increasing brain lesion load and brain atrophy have also been found to correlate with the progression of cognitive impairment in MS (4). Indeed, changes in brain gray matter—rather than the white matter—have been shown to predict long-term physical disability and cognitive impairment in a number of studies (5–8). A review by Langer-Gould et al, though, identified sphincter symptoms as the most robust predictor of long-term physical disability (9). More recently, deep gray matter alterations and in particular thalamic atrophy have gained increasing relevance in the study of MS. For example, a study on subjects with radiologically isolated syndrome (RIS) has provided evidence that thalamic atrophy may precede clinical manifestations of CNS demyelination, therefore suggesting the thalamus may be a key region to check for early signs of neurodegeneration in MS (10). Furthermore, thalamic atrophy has also been found to correlate with cognitive decline and disability, suggesting that thalamic volume may be a clinically relevant biomarker to assess the neurodegenerative disease process in MS (11, 12).

From a functional point of view, studies using task-related functional MRI (fMRI) have often demonstrated greater responses in cortical areas, particularly in early stage MS patients, when compared with healthy controls. These differences are generally interpreted as evidence of compensatory mechanisms to ameliorate cognitive or sensorimotor deficits in the initial stages of the disease (13–17). Together with altered functional connectivity between brain regions during cognitive tasks, such effects imply the use of brain reserve to limit cognitive impairment (18). Increased functional connectivity (FC) in MS has also been reported in task-free conditions, that is, resting state functional MRI (rs-fMRI). One longitudinal rs-fMRI study reported that increased FC was detected after the advent of new lesions, which was interpreted as an attempt to compensate for tissue damage (19). It remains to be verified whether such a functional reorganization leads to a preservation of wellbeing. For example, an increased FC in clinically isolated syndrome (CIS) patients without conventional lesions has been suggested as a risk factor for MS (20). Interestingly, recent studies in Relapsing Remitting MS (RRMS) have reported a positive correlation between increased FC in thalamic or in fronto-parietal regions and fatigue scores, suggesting that increased FC might be a maladaptative process (21, 22). Other studies have reported evidence of positive correlation between areas of increased FC and structural damage (23) or have found an association between increased functional connectivity in distinct systems involving attention and cognitive control with decreased cognitive ability at early stages of MS (24), challenging the concept of functional compensation in MS. Nevertheless, a recent study of Rocca et al. showed that also the reverse condition is possible, reporting the evidence of reduced FC correlated with better neuropsychological performance in a large cohort of MS subjects (25), furtherly questioning the interpretation of altered FC in MS.

An understanding of brain function in MS may be better served by looking across the many functional networks in the brain, as the diffuse brain injuries present in MS are best revealed when co-varying fluctuations of the blood-oxygen-level-dependent (BOLD) signals are identified across widely dispersed neural structures (26). These networks are most readily evident during periods of minimal cognitive demand, that is, when rs-fMRI is used to reveal resting state networks (RSNs). These RSNs engage distinct brain regions that exhibit unique spontaneous patterns of low-frequency (around 0.01–0.1 Hz) synchronisations and by inference functional connectivity (FC) (27, 26). Looking at resting state is particularly suited for disorders such as MS in which individuals may show cognitive impairments. For example, the default mode network (DMN) is a RSN that has particular relevance as a surrogate marker for early dementia (28, 29). Examination of rs-fMRI has provided important insights into the functional reorganization of the brain in subjects with early relapsing MS (3–5 years disease duration) (30) as well as in MS subjects at more advanced disease stage (31) or with longer disease duration (32).

In this study, we used an advanced rs-fMRI approach to focus on network changes associated with radiological and clinical scores. First of all, we performed a traditional analysis to see which RSNs are affected by RRMS in a cohort of patients with mild impairments. Then, given that MS can be described as a multisystem disconnection syndrome (33), this work performed a comprehensive advanced analysis of the functional status of the principal large-scale RSNs focusing on identifying patterns of RSN FC impairment that discriminate mild RRMS from healthy subjects. To better understand the nature of the detected FC alterations we formulated *a priori* hypotheses of mechanisms based on FC correlations with radiological and clinical metrics. We also compared RRMS subjects with short disease duration (MS_short_ or early MS) with those with longer disease duration (MS_long_ or established MS) to assess the impact of disease duration on FC.

## Materials and methods

### Subjects

MRI acquisitions were performed on 91 subjects. Based on the McDonald criteria (34) 62 subjects with RRMS (age 38.58 ± 8.25, MSSS = 2.89 ± 1.87) were recruited for the study from the Christchurch Hospital (Christchurch, New Zealand). The twenty-nine healthy controls (HC) aged 34.45 ± 10.17 years had no previous history of neurological disorders. All MS patients had been relapse free and clinically stable for at least 1 month before study entry and 10 were receiving disease modifying medications. Neurological, neuropsychological and MRI assessments were scheduled over 1 month in 3 visits. Neurological findings not attributable to MS and psychiatric symptoms (e.g., cerebrovascular disease, tumors, brain surgery, depressive disorder as measured by Beck Depression Inventory (BDI) with BDI > 19 cut-off) were defined as exclusion criteria. The RRMS group was also subdivided (labeled MS_short_ and MS_long_) based on their disease duration (35). The MS_short_ group comprised 36 subjects with early RRMS (defined as ≤5 years from symptom onset, aged 37.34 ± 8.82, MSSS = 2.95 ± 1.99). The MS_long_ group included 26 subjects with a more established RRMS disease duration (between 5 and 15 years from symptom onset, aged 40.62 ± 7.26, MSSS = 2.86 ± 1.79). All subjects received an MRI scan and clinical assessment by a multidisciplinary team at the New Zealand Brain Research Institute (NZBRI). The study was approved by the Lower South regional ethics committee of New Zealand and written informed consent was provided by all participants.

### Clinical-neurological assessment

All patients underwent clinical assessment, including relapse history, Expanded Disability Status score (EDSS) (36), and Modified Fatigue Impact Scale (MFIS) (37). MS severity score (MSSS) (38) was calculated for all patients. Patients were assessed for depression using the Beck Depression Inventory (BDI-II) (39), while subjects' premorbid IQ was estimated with the Wechsler Test of Adult Reading (WTAR) (40). All participants performed the Montreal Cognitive Assessment (MoCA) (41) and 11 standard neuropsychological tests covering four cognitive domains: executive function (letter fluency, category fluency, Stroop interference) (42), memory (episodic learning and recall were assessed (both visual) with the Brief Visual Memory Test, BVMT) (43), attention and working memory [Stroop colors, word reading, Symbol Digit Modality Test (SDMT), Paced Auditory Serial Addition Test (PASAT)] (44), and visuospatial function [Judgment of Line Orientation (45), Rey Complex Figure copy (46)]. All patients were also assessed using the MS Functional Composite (MSFC) test (47). MSFC score was calculated from three components: (i) the average scores from the four trials on the 9-HPT, (ii) the average scores of two 25-Foot Timed Walk trials and (iii) the number correct from the PASAT-3. Raw test scores were converted to z-scores using age-adjusted and gender-adjusted normative data for each test and then averaged for each domain.

### MRI acquisition

All scans were acquired in a single session on a 3T General Electric Signa HDxt MR scanner (General Electric Medical Systems, Milwaukee, WI) with head coil.

All subjects underwent MRI examination that included:

- rs-fMRI: T2^*^ Gradient Echo (GRE), echo planar imaging (EPI) sequence (TR/TE = 2500/35ms; voxel size = 3.75 × 3.75 × 4 mm^3^, FOV = 240 mm, 37 slices, 240 volumes, acquisition time = 10:10 min). During fMRI acquisition subjects were asked to keep their eyes open while fixating on a cross; this method may improve reliability relative to “eyes closed” (48).
- T1 volumetric imaging (for anatomical reference): 3D T1-weighted inversion-prepared spoiled gradient recalled-echo acquisition (IR-SPGR): TR/TE = 2.8/6.6 ms, TI = 400 ms; flip angle = 15°, acquisition matrix = 256 × 256 × 180; reconstruction matrix = 512 × 512 × 180 FOV = 240 mm; voxel size = 0.48 × 0.48 × 1 mm^3^, 180 slices) was acquired for anatomical reference.

Conventional MRI sequences were also acquired for lesion detection:

- T2 Flair Spin-Echo (SE): TE/TR = 11/500 ms, TI = 2250 ms, FOV = 220 mm, voxel size = 0.43 × 0.43 × 3 mm^3^.
- T2 Propeller: SE, TE/TR = 98/3700 ms, FOV = 220 mm, voxel size = 0.43 × 0.43 × 3 mm^3^.
- T1 SE: TE/TR = 12/500 ms, FOV = 220 mm, voxel size = 0.43 × 0.43 × 3 mm^3^.

### Structural MRI analysis

#### Lesion load evaluation and lesion filling

For each subject, MS lesions were manually outlined using Jim software (Jim 4.0 Xinapse System Leicester, UK) on T2 Flair images to quantify T2 lesion load (49). MS lesions were also manually outlined on 3D T1-weighted (3D T1) images and filled using an automatic lesion filling program (LEAP) (50) before performing tissue segmentation procedures in order to limit potential gray matter (GM) and white matter (WM) misclassification due to signal abnormalities in the lesion tissue.

#### Tissue segmentation analysis

For each subject, GM and WM volumes as well as the total intracranial volume were obtained performing tissue segmentation on 3D T1 images using SPM8 (Statistical Parametric Mapping, Wellcome Department of Imaging Neuroscience Group, London, UK). For each subject, after lesion filling, 3D T1 images were intensity bias corrected, tissue classified and registered using linear and non-linear transformations (DARTEL) within a unified model (51). The resulted images were then segmented into GM, WM, and cerebrospinal fluid (CSF) using the customized priors, masked to remove non-brain tissue voxels, modulated, and finally smoothed with a 10 mm Gaussian kernel (49). For the purposes of the study, GM volume was calculated in subject space and divided by the total intracranial volume—defined as the sum of GM, WM, and CSF segments—in order to obtain a normalized GM volume index.

### rs-fMRI analysis

For each subject, rs-fMRI images were analyzed using the Independent Component Analysis (ICA) first at single-subject pre-processing level (single-ICA) to reliably separate signal from noise, using the ICA-based Xnoiseifier (FIX) tool (52) as implemented in FSL (FMRIB Software Library, version 5.0.9). ICA was then applied at group-level (group-ICA) on pre-processed rs-fMRI data using the Multivariate Exploratory Linear Optimized Decomposition into Independent Components (MELODIC) method in order to characterize the RSNs (53).

#### Data pre-processing

Individual subjects' pre-processing was performed using FSL tools and consisted in motion correction, brain extraction, spatial smoothing using a Gaussian kernel of full-width-at-half-maximum (FWHM) of 5 mm, and high pass temporal filtering equivalent to 150 s (0.007 Hz). Individual rs-fMRI volumes were than registered to the corresponding structural 3D T1 scan using FMRIB's Linear Image Registration Tool (FLIRT) and subsequently to standard space (MNI152) using FMRIB's Nonlinear Image Registration Tool (FNIRT) with default options. Then, each 4-dimensional rs-fMRI dataset entered single-subject spatial-ICA (single-ICA) decomposition using MELODIC, with an automatic estimation of the number of independent components (ICs), which resulted in spatial maps, each with an associated time course. Model order was estimated using the Laplace approximation to the Bayesian evidence for a probabilistic principal component model. For each subject, single-ICA results were finally processed with the FIX algorithm to clean rs-fMRI data from noisy and artefactual components.

#### RSNs identification

Pre-processed functional data, containing 240 time points (volumes) for each subject, were temporally concatenated across subjects to create a single 4-dimensional data set to run the group-ICA analysis via MELODIC, with an automatic estimation of the number of ICs. At this level, some of the ICs were identified as noise while others as RSNs, based on previous literature (53–56). Group-ICA decomposes data into spatial maps that are the ICs relative to the total processed dataset (i.e., the enrolled 91 subjects), or the multi-subject ICA components. At group level, the IC maps are the same for each subject and are used as inputs for the subsequent dual regression analysis in order to calculate the statistical inference among groups.

#### Between group RSNs comparison and global alterations ranking

A non-parametric permutation test, referred to as “dual regression” (28, 57, 58), was then applied to compare group-specific FC maps for each IC map. First, this analysis tested the statistical differences between HC and MS using two comparisons or contrasts (MS < HC and MS > HC). We then investigated the presence of significant differences in RSN FC between MS_short_ and MS_long_ subjects, by directly testing the MS subgroups with two further contrasts: MS_short_ >MS_long_ and MS_short_ < MS_long_.

In this study, each dual regression analysis was carried out on the total ICs using age, gender, education level and GM ratio as additional covariates included in the general linear model (GLM). The statistical inference at group level was performed using 5000 permutations. The resulting statistical maps were family-wise error (FWE) corrected for multiple comparisons, implementing threshold-free cluster enhancement (TFCE) (59) using a significance threshold of at least *p* ≤ 0.05. After that, the final statistical maps were saved as *tstatFC* maps.

In order to study the FC changes within each RSN and to establish a ranking of the networks in terms of their alterations, for each considered contrast we calculated a global parameter, referred to as *global FC* or *gFC* (29) which takes into account both the extension of the clusters and the magnitude of the FC changes. For each contrast, we used the *gFC* index only to produce a bar plot that ranked and compared the RSNs in terms of their functional alteration (i.e., decreasing/increasing *gFC*-values), taking into account both the magnitude and the spatial extent of their FC changes.

#### RSNs correlations with lesion load and MSSS

FC changes have been then correlated with the radiological T2LL score and subsequently with the clinical MSSS index, which are clinically relevant for MS diagnosis. For this analysis, we started using the voxels surviving the FWE-corrected threshold (*p* ≤ 0.05) in the resulting *tstatFC* maps (each of MS < HC and MS > HC). We then used these masks to run a second permutation analysis using T2 lesion load (T2LL) as the explanatory variable of interest in the design matrix of the GLM (60). The new resulting *tstat* maps were saved as *tstatFC*_*T*2*LL*_ and included only those RSN voxels that were both FC altered and significantly (FWE-corrected *p* ≤ 0.05) correlated to T2LL. We considered non-null areas within the *tstatFC*_*T*2*LL*_ maps to calculate parameter estimates, as expressed by *Z*-values in individual masked rs-fMRI images, to obtain a numerical value of the strength of RSNs temporal coherence.

In order to assess whether the alterations in the *tstatFC*_*T*2*LL*_ maps might correlate with MSSS, we used the *tstatFC*_*T*2*LL*_ maps as masks to run a third permutation analysis with MSSS as explanatory variable of interest. The resulting statistical maps were saved as *tstatFC*_*T*2*LL*_*MSSS*_ and included only the areas within RSNs that were FC altered, significantly correlated (FWE-corrected *p* ≤ 0.05) to T2LL and MSSS.

Pearson's correlation analysis was carried out using SPSS to obtain a numerical value of the correlation strength between RSN FC change and T2LL for the non-null areas in the *tstatFC*_*T*2*LL*_ maps and between RSN FC change + T2LL and MSSS in the *tstatFC*_*T*2*LL*_*MSSS*_ maps.

#### Mechanisms of FC alterations

In order to facilitate the discussion on the mechanisms of FC alterations in RRMS we introduced *a priori* a method of analysis to help interpreting possible scenarios, as outlined in Table 2 and in the flowchart diagram of Figure S1 in Supplementary Material. It is known that FC can be found both increased or decreased in MS compared to HC (61), but interpretation of such changes is debated. By analysing possible correlations between FC changes and T2LL it may be possible to hypothesize mechanisms of such changes. Specifically, we looked for correlations between FC and T2LL and searched the data for four possible scenarios: (1) increased FC correlating with lower T2LL; (2) decreased FC and higher T2LL; (3) increased FC and higher T2LL; (4) decreased FC and lower T2LL. While the first two scenarios are straightforward (see discussion), interpretation of scenarios where T2LL and FC go in the same direction are less intuitive. For this reason, we performed a further correlation analysis with the MSSS and defined the following four further scenarios: (3a) increased FC, higher T2LL and lower MSSS; (3b) increased FC, higher T2LL, and higher MSSS; (4a) decreased FC, lower T2LL, lower MSSS; (4b) decreased FC, lower T2LL, higher MSSS.

Areas identified as having different *FC*-values between MS_short_ and MS_long_ were also classified in comparison with the above table of differences between the entire MS cohort and HC.

### Non-imaging statistics

Statistical analyses were carried out using SPSS (version 21.0; SPSS, Chicago, IL, USA). Demographic, behavioral and radiological differences between groups were assessed with different tests depending on the typology of the variables (binary, normally or non-normally distributed). Specifically, χ^2^-test was performed to compare frequency distributions of gender in the three groups. One-way analysis of variance (ANOVA) with Bonferroni correction was used to assess statistical differences among groups (HC and MS; HC, MS_short_ and MS_long_) in age. Non-parametric Kruskal-Wallis test was applied to test differences among the groups in education level, clinical indices (WTAR, BDI and MSFC, see section Clinical-Neurological Assessment for details) and neuropsychological scores (MoCA, PASAT, and attention, memory, executive, visuospatial cognitive domains). Non-parametric Mann-Whitney *U*-test was performed to test differences between MS_short_ and MS_long_ groups in EDSS, MSSS, MFIS, disease duration and lesion load (T2LL). A Pearson's correlations analysis was performed to assess the association between the RSN FC change and T2LL for the non-null areas in the *tstatFC*_*T*2*LL*_ maps and between RSN FC change + T2LL and MSSS in the *tstatFC*_*T*2*LL*_*MSSS*_ maps (see section RSNs Correlations With Lesion Load and MSSS for details). Results were Bonferroni corrected for multiple comparisons and a statistical threshold of *p* ≤ 0.05 was considered significant.

## Results

### Clinical and neurological characteristics

The demographic and clinical scores for the HC, MS, and MS_short_ and MS_long_ subgroups are provided in Table 1. Except for MoCA, both MS groups performed worse than HC on all clinical and neuropsychological measures. A significant difference was found for age between HC and MS (*p* = 0.05) and in particular between HC and MS_long_ subjects (*p* = 0.041), with MS_long_ group older than HC. Significant differences were found in disease duration (*p* < 0.001) and EDSS score (*p* = 0.008) when comparing MS_short_ and MS_long_ groups, with higher EDSS scores observed in MS_long_. The mean MSFC score was significantly reduced in both MS_short_ and MS_long_ compared with HC (Mann-Whitney test, MS_short_: *p* = 0.003; MS_long_: *p* = 0.012), but no significant differences were observed in MSFC between MS_short_ and MS_long_. Both MS groups also had significantly higher BDI scores (Mann-Whitney test, MS_short_: *p* = 0.008; MS_long_: *p* = 0.003 than HC. MFIS, MSSS, and T2 lesion load (i.e., T2LL) were not significantly different between MS_short_ and MS_long_ (measures not relevant for HC).

**Table 1:** Demographic and clinical characteristics.

	**HC (*n* = 29)**	**MS (*n* = 62)**	**MS_short_ (*n* = 36)**	**MS_long_ (*n* = 26)**	***p***
Gender (female/male)	21/8	47/15	30/6	17/9	> 0.2
Age (years)	34.45 ± 10.17	38.58 ± 8.25	37.34 ± 8.82	40.62 ± 7.26	< **0.05**^a^^,^ ^c^
Education (years)	13.62 ± 2.19	13.03 ± 2.53	13.43 ± 2.73	12.38 ± 2.17	> 0.05
WTAR	107.62 ± 7.08	104.65 ± 9.40	104.29 ± 9.90	105.15 ± 9.05	> 0.1
Disease duration (years)	n.a.	5.27 ± 4.08	2.29 ± 1.22	9.46 ± 2.80	< **0.001**^d^
EDSS	n.a.	1.77 ± 1.18	1.42 ± 0.90	2.23 ± 1.40	**0.008**^d^
MSSS	n.a.	2.89 ± 1.87	2.95 ± 1.99	2.86 ± 1.79	> 0.05
MFIS	n.a.	7.45 ± 4.66	7.69 ± 4.95	7.15 ± 4.53	> 0.1
BDI	4.45 ± 5.24	8.32 ± 6.10	8.29 ± 6.90	8.23 ± 5.20	< **0.05**^a^^,^ ^b^^,^ ^c^
PASAT (z score)	0.21 ± 1.05	0.62 ± 1.22	0.56 ± 1.07	0.70 ± 1.44	> 0.1
SDMT (z score)	0.29 ± 1.03	0.12 ± 0.91	0.04 ± 0.92	0.21 ± 0.93	> 0.2
MSFC	0.36 ± 0.46	0.11 ± 0.70	0.04 ± 0.58	0.18 ± 0.86	< **0.05**^a^^,^^b^^,^ ^c^
MoCA	28.68 ± 1.51	28.29 ± 1.76	28.17 ± 1.82	28.46 ± 1.72	> 0.2
Executive (z score)	0.80 ± 0.54	0.39 ± 0.77	0.46 ± 0.76	0.28 ± 0.81	> 0.05
Memory (z score)	0.71 ± 0.68	0.47 ± 0.79	0.43 ± 0.84	0.50 ± 0.74	> 0.3
Attention (z score)	0.02 ± 0.71	0.32 ± 0.71	0.29 ± 0.76	0.36 ± 0.66	> 0.1
Visuospatial (z score)	0.27 ± 0.55	0.04 ± 0.59	0.13 ± 0.52	0.09 ± 0.68	> 0.05
Composite z score	0.44 ± 0.49	0.18 ± 0.53	0.22 ± 0.53	0.12 ± 0.68	> 0.05
T2 lesion load (mL)	n.a.	16.63 ± 22.23	16.49 ± 23.77	16.82 ± 20.84	> 0.1

WTAR, Wechsler Test of Adult Reading; EDSS, Expanded Disability Status score; MSSS, MS severity score; MFIS, Modified Fatigue Impact Scale; BDI, Beck Depression Inventory; PASAT, Paced Auditory Serial Addition Test; SDMT, Symbol Digit Modality Test; MSFC, MS Functional Composite; MoCA, Montreal Cognitive Assessment. Mean and SD are reported. A chi-square test was used to test difference in gender, whereas one-way ANOVA test was used to test difference in age. Non-parametric Kruskal-Wallis and Mann-Whitney tests were used to test all the other measures. Significant findings are shown in bold.

a *Significant difference between HC and MS*.

b Significant difference between HC and MS_short._

c Significant difference between HC and MS_long._

d Significant difference between MS_short_ and MS_long._

### RSNs identification

ICA processing on rs-fMRI images resulted in 35 independent components, 18 of which were classified as RSNs based on their frequency spectra and spatial patterns (29, 53, 54, 56). The remaining 17 components probably reflected artifacts like movement, physiological noise or cerebro-spinal fluid (CSF) partial volume effects (62).

The identified 18 RSNs were: medial visual network (*MVN*), lateral visual network (*LVN*), precuneus network (*PN)*, superior precuneus network (*PNsup*), sensory motor networks area M1 (*SMNm1)*, and area S2 (*SMNs2*), auditory network (*AN*), executive control network (*ECN*), default mode network (*DMN*), anterior default mode network (*DMNa*), frontal cortex network (*FCN*), language networks (*LN*) anterior (*a*) and posterior (*p*), right (*R*) and left (*L*) ventral attention networks (*VAN*), salience network (*SN*), task positive network (TPN) and cerebellar network (*CBLN*). The cortical regions associated with identified RSNs are provided as Supplementary Material (Figure S2).

### MS vs. HC: RSNs comparison and ranking of the RSN alterations

The analysis of FC within the 18 identified RSNs revealed that 16 networks, including MVN, LVN, PN, PNsup, SMNm1, AN, ECN, DMN, DMNa, FCN, LNa, LNp, LVAN, SN, TPN, and CBLN, were functionally impaired in MS compared to HC. Only RVAN and SMNs2 did not show any significant FC impairment when comparing MS to HC.

**Figure 1:**
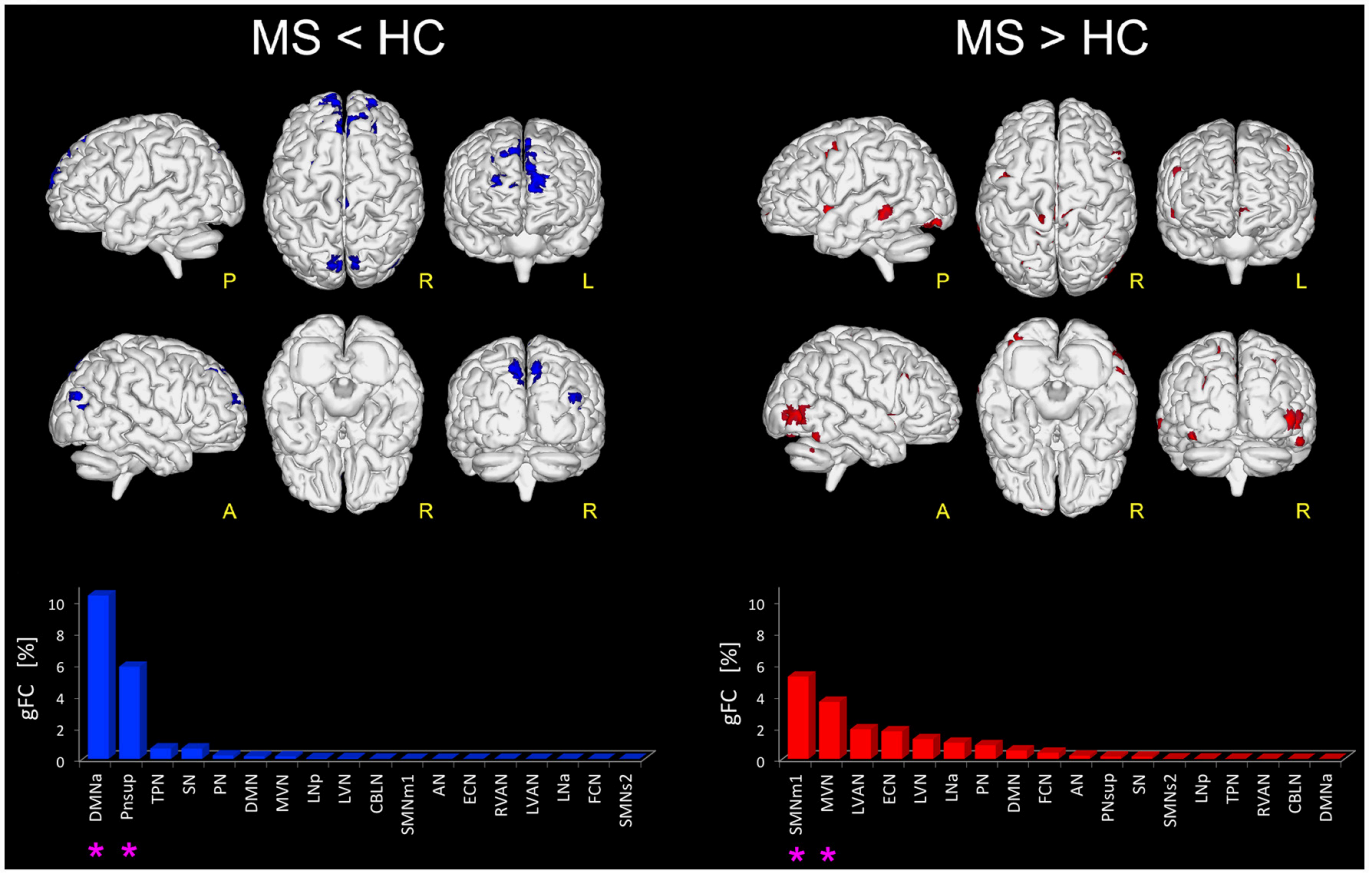
Altered FC in RSNs of MS vs. HC. On the left: in blue, brain areas showing significantly reduced FC (*p* ≤ 0.01 FWE-corrected) within the RSNs in MS compared to HC (i.e., MS < HC). The blue bar plot on the bottom shows, for MS < HC, the ranking of the RSNs according to their *gFC* alteration: DMNa and PNsup (highlighted with an asterisk mark in the bar plot) resulted the networks with the largest FC reductions in MS. On the right: global map showing on top, in red, the RSN voxels that resulted to have a significantly increased FC (*p* ≤ 0.01 FWE-corrected) in MS vs HC (i.e., MS > HC). The details of each RSN alteration for MS > HC are reported in the red bar plot on the bottom right: SMNm1, MVN (highlighted with an asterisk mark in the bar plot) resulted as the top-ranked altered networks.

When looking at the global profile of FC impairments resulting from the group analysis, large (more than 1000 voxels) significantly FC reduced (*p* < 0.01) areas in MS compared to HC (i.e., MS < HC contrast) were observed in the frontal cortex, mainly involving the medial frontal gyrus of DMNa, and the precuneus area of PNsup and TPN (Figure 1). Decreased FC areas were also found in the anterior cingulate cortex (BA10) and the fusiform gyrus (BA19) involving SN. Coherent results were found when looking at the order ranking of RSNs according to the *gFC* index, the DMNa and PNsup networks showing the largest and most severe FC reductions in MS (Figure 1).

Furthermore, large areas of significantly increased FC (*p* < 0.01) were observed in MS group compared with HC (i.e., MS > HC) in the right supplementary motor area, cingulum, right fusiform gyrus and the most anterior part of the precuneus, mainly involving SMNm1 (Figure 1). Extended areas of increased FC were also found in the inferior and middle occipital gyri of MVN. Further areas of increased FC were detected in the left middle temporal gyrus, mainly involving LVAN, as well as in both left and right insula areas and in the frontal middle gyrus of ECN. Coherent results were found even when considering the *gFC* network ranking that highlighted SMNm1 and MVN as the most affected networks for the MS > HC contrast (Figure 1).

### Correlations between FC changes, T2LL and MSSS

**Figure 2:**
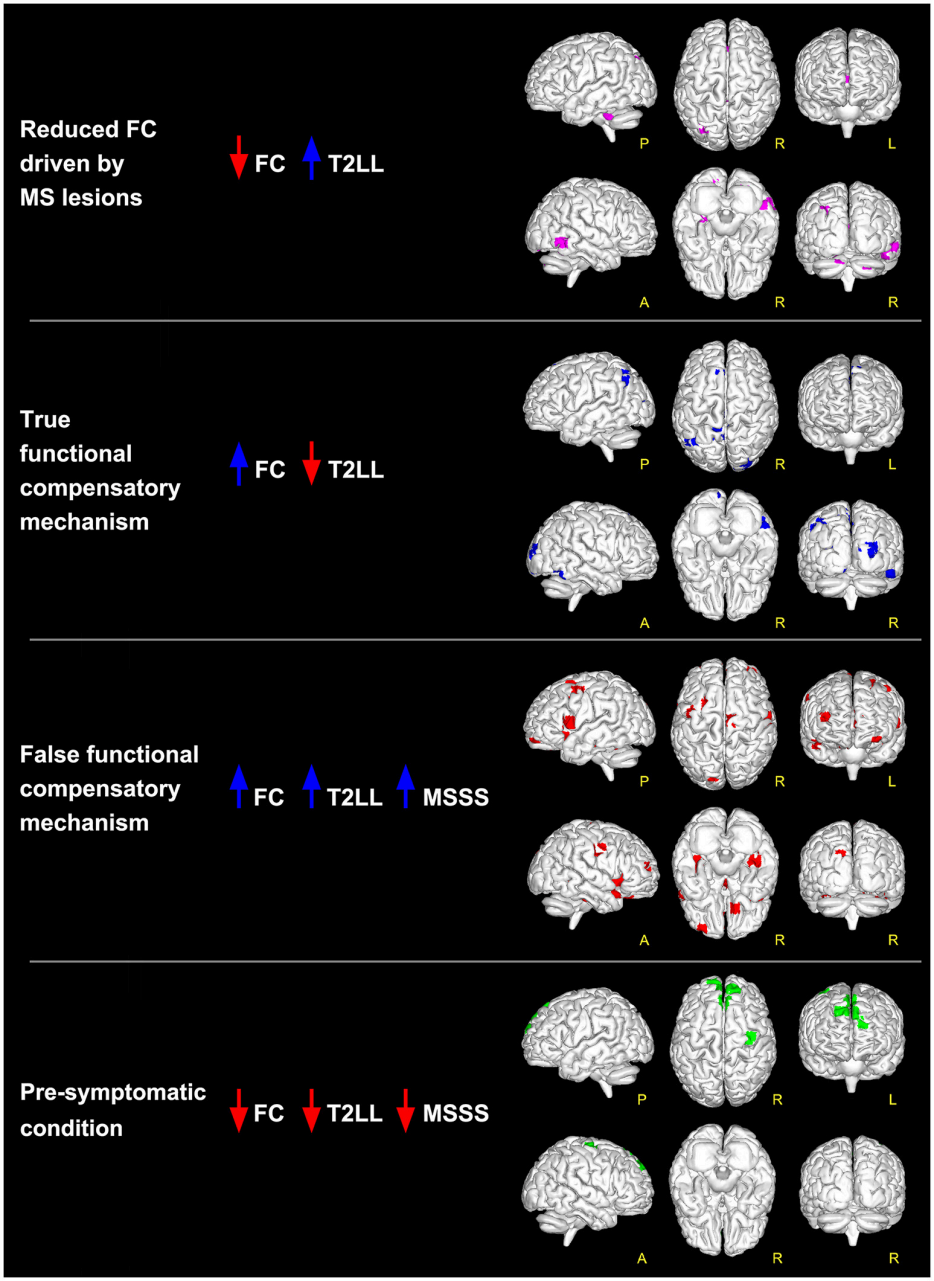
Global maps of FC alterations which correlate with the overall lesion load (T2LL) and MSSS. Findings have been interpreted according to the multiple-scenario hypothesis presented in Table 2: four of the six proposed mechanism have been identified and represented as a map: (1) *reduced FC driven by lesion (magenta* voxels, corresponding to reduced FC areas in MS that negatively correlated at *p* ≤ 0.05 FWE-corrected with T2LL); (2) *true functional compensation (blue* voxels, corresponding to increased FC areas in MS that negatively correlated with T2LL); (3) *false functional compensation (red* voxels, corresponding to increased FC areas in MS that positively correlated with T2LL and MSSS); (4) *pre-symptomatic condition (green* voxels, corresponding to decreased FC areas in MS that positively correlated with T2LL and MSSS).

When comparing MS to HC, results show that there are at least 4 possible combinations of correlations between T2LL and FC and MSSS in a number of areas of the brain (see Figure 2 for a visual description of these findings). These can be also linked to scenarios depicted in Table 2:

1. Low FC and high T2LL (Table 2: Scenario 1): Areas of reduced FC in MS compared to HC were found to correlate negatively with T2LL in MVN (posterior cerebellar declive), PN (right posterior cingulate cortex, BA19), CBLN (left cerebellar lobule VI), LVN (left cerebellar Crus I), SN (right inferior and medial temporal gyrus, BA37), and TPN (right cerebellar Crus I).
2. High FC and low T2LL (Table 2: Scenario 2): Areas of increased FC in MS compared to HC were found to correlate negatively with T2LL in areas of MVN (left calcarine and cuneus, BA30), SMNm1 (left precuneus, BA7), ECN (left superior and medial frontal gyrus), LVAN (left angular gyrus), and LVN (right superior occipital gyrus and cuneus, BA18, BA19).
3. High FC and high T2LL (Table 2: Scenario 3a or b): Areas of increased FC in MS compared to HC were found to correlate positively with T2LL in MVN (left superior occipital gyrus and cuneus, BA19), PNsup (right precuneus), AN, ECN (left anterior cingulate cortex, BA10), LVAN (BA40), LNa (left precentral gyrus, BA44) and FCN (BA11). Of these areas, those in MVN, ECN, LNa and FCN showed also positive correlations with MSSS (Table 2: Scenario 3b).
4. Low FC and low T2LL (Table 2: Scenario 4a or b): Areas of reduced FC in MS compared to HC were found to correlate positively with T2LL in DMNa (right superior and medial frontal gyri) and TPN (right precentral gyrus, BA6). These areas were also found to positively correlate with MSSS (Table 2: Scenario 4b).

**Table 2:** Proposed analysis and hypothesis of mechanisms of functional connectivity (FC) alterations in MS.

**Scenario**	**Analysis**			**Hypothesis of mechanism**
	**FC**	**T2LL**	**MSSS**	
Scenario 1	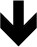	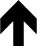		FC reductions driven by MS lesions
Scenario 2	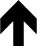	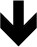		True functional compensation
Scenario 3a	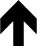	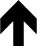	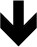	Functional compensation attempt
Scenario 3b	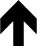	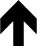	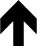	False functional compensation
Scenario 4a	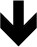	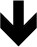	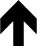	Neurodegeneration (reduced FC not due to MS lesions)
Scenario 4b	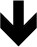	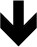	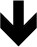	Pre-symptomatic condition (damage precedes clinical manifestation of MS)

*Multiple-scenarios have been hypothesized to interpret the role of FC changes within the resting state networks (RSNs) of MS subjects. Each proposed mechanism describes a specific relation between FC changes, overall lesion load (T2LL) and MS severity score (MSSS)*.

### MS_**short**_ vs. MS_**long**_: RSNs comparison and ranking of the RSN alterations

**Figure 3:**
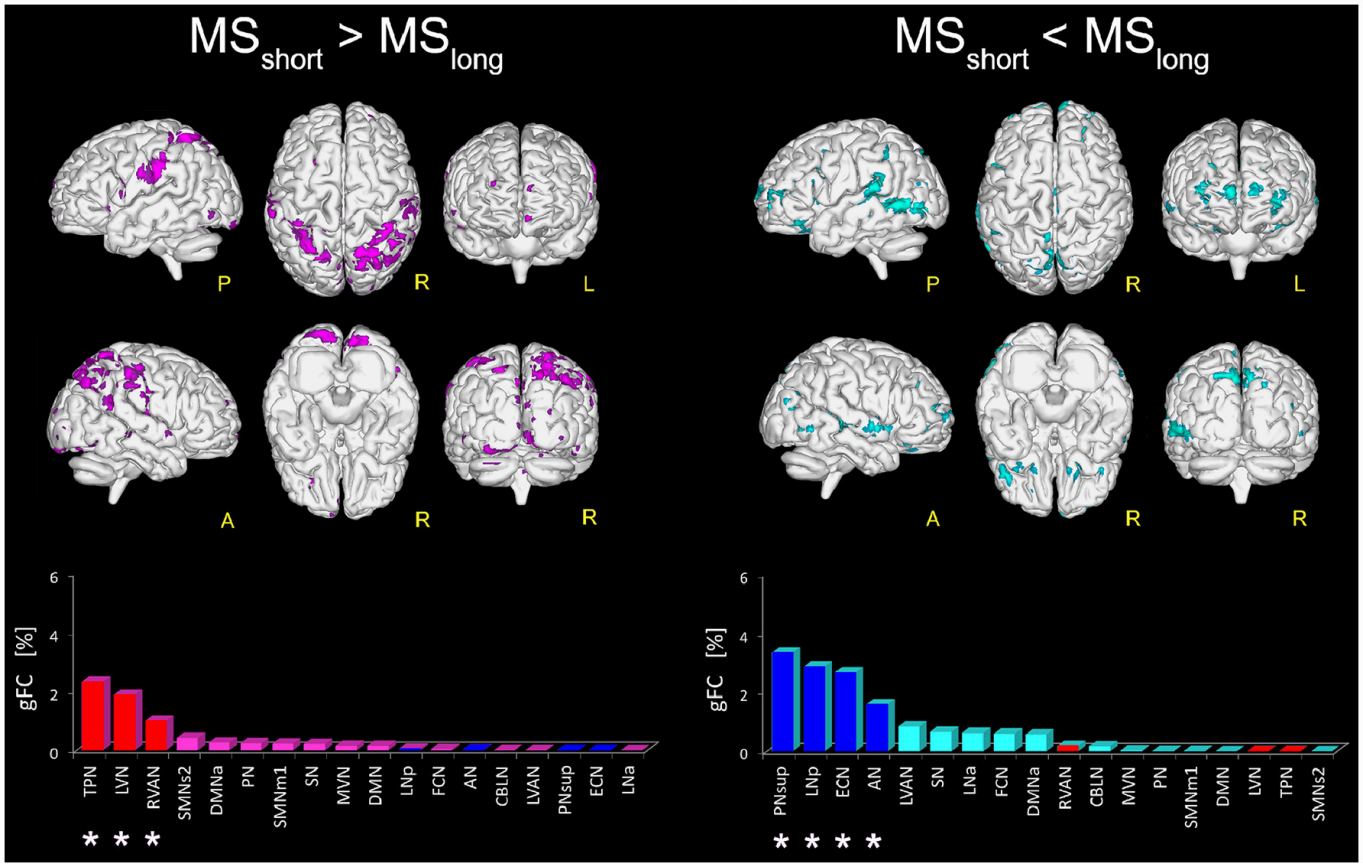
Areas with altered FC in RSNs of MS_short_ compared to MS_long_ patients. On the left: magenta voxels show the areas of significantly greater FC (*p* ≤ 0.01, FWE-corrected) in the MS_short_ > MS_long_ contrast. On the right: aquamarine voxels represent the global map of the areas found with significantly lower FC (*p* ≤ 0.01, FWE-corrected) in MS_short_ compared to MS_long_ (i.e., MS_short_ < MS_long_). Interestingly, the direct comparison of MS_short_ and MS_long_ highlighted a distinct pattern of FC differences. Below the brain representations of areas of differences, bar plots show, for each considered contrast, the ranking of the RSNs according to their *gFC* parameter. In each bar plot we colored in red the top-ranked networks for MS_short_ > MS_long_ and in blue the top-ranked networks for the MS_short_ < MS_long_ contrast. Note that the top-ranked networks (marked with an asterisk) in one contrast (e.g., MS_short_ > MS_long_) are also some of the bottom-ranked network in the opposite contrast (MS_short_ < MS_long_).

Direct comparison of the MS_short_ and MS_long_ groups revealed several areas of significantly greater FC (*p* < 0.01) in MS_short_ (i.e., MS_short_ > MS_long_) both in left and right parietal areas of the supramarginal gyrus, right precuneus, thalamus, and posterior cingulate cortex, mainly involving the TPN, LVN, and RVAN (Figure 3). The *gFC* network ranking reported coherent results, showing TPN, LVN, and RVAN as the top-ranked RSNs with a different gFC for the MS_short_ > MS_long_ contrast. Overlapping these areas onto the maps of alterations corrected for T2LL and MSSS from the whole MS group compared to HC (Figure 2), 4.36% of the greater FC in MS_short_ > MS_long_ corresponds to regions interpreted as *true functional compensation* areas (Table 2: Scenario 2) in the precuneus and in the superior frontal gyrus. Only 0.3 and 0.1% of the altered regions in MS_short_ > MS_long_ overlap respectively with *reduced FC driven by lesions* (Table 2: Scenario 1) in the anterior cingulum and with areas interpreted as evidence of *pre-symptomatic condition* (Table 2: Scenario 4b) in the medial frontal gyrus.

When considering the MS_short_ < MS_long_ contrast, areas of significantly reduced FC (*p* < 0.01) in MS_short_ compared to MS_long_ were observed in the left middle cingulum and the right precuneus and fusiform gyrus (BA37) of PNsup, in the right inferior occipital gyrus (BA19) of LNp, in the middle frontal gyrus of ECN as well as in both right and left middle temporal gyrus of AN (Figure 3). PNsup, LNp, followed by ECN and AN, also resulted as the most *gFC* altered networks in MS_short_ < MS_long_ (Figure 3). Moreover, when overlapping these areas to areas of alterations corrected for T2LL and MSSS at whole group level (Figure 2), 1.2% corresponds to regions of *true functional compensation* (Table 2: Scenario 2) in the cingulate gyrus, while 0.5% overlaps with areas of *reduced FC driven by lesions* (Table 2: Scenario 1) in the inferior temporal gyrus. Only 0.2 and 0.02% of the altered regions in MS_short_ < MS_long_ overlap respectively with *pre-symptomatic condition* (Table 2: Scenario 4b) in the superior frontal gyrus and with *false functional compensation* (Table 2: Scenario 3b) in the inferior frontal gyrus.

## Discussion

In the current study, we used ICA and dual regression techniques to investigate whether and how functional connectivity within the RSNs is affected by the disease in a mild cohort of RRMS subjects. Results confirm a widespread functional alteration, expressed as both areas of decreased and increased FC, of almost all the RSNs, compared to HC. This result supports the interpretation of MS as a multisystem disconnection syndrome (33). Compared to HC, RRMS subjects show decreased FC in two RSNs: DMNa and PNsup, both of which are cognitive networks involved in high cognitive functioning such as working memory, memory retrieval, and future-oriented thinking (63). FC reductions within the DMN and precuneus in RRMS patients has been reported previously (64, 65) and ascribed to factors, such as brain hypometabolism and hypoperfusion. The mechanisms of FC alterations are still debated and future multi-modal and longitudinal studies should aim to pin down the origin of such damage.

Our results highlight that, compared to HC, RRMS subjects have increased FC involving two different RSNs: SMNm1, which play a role in motor-control functioning, and MVN, which is involved in visual and language functions (66). Evidence of increased FC within the RSNs of MS patients' brain has now been observed in multiple studies with and without the presence of conventional lesions (19, 20, 67, 68). Taken together these studies support the hypothesis that increased FC may be a beneficial compensatory mechanism occurring at least at early stages of MS (69) which is lost in more advanced disease (31). Interestingly, the same pattern of increased FC linked to white matter (WM) damage, followed by a subsequent global FC reduction has been demonstrated using an empirical model by Tewarie et al. (70). FC increase has also been linked to cognitive reserve and the ability of one's brain to adapt and delay cognitive decline (18, 71). Nevertheless, the hypothesis that an increased FC can be considered as evidence of brain functional reorganization processes (either beneficial or maladaptive) is still to be established (72, 73).

In order to help fostering novel discussions on this topic we propose to analyse FC changes in relation to other pathological markers. Given the specificity of demyelinating lesions to MS, their diagnostic role and their long term predicted value, we believe that T2LL is an important factor to be studied in association with FC changes, at least in the first instance. Furthermore, a clinical score like the MSSS can introduce evaluation of disease severity that encompasses both EDSS and disease duration. Other factors could be equally considered in alternative models to the one that we proposed, such as thalamic atrophy form longitudinal data, which has been suggested as a potentially relevant biomarker to assess the neurodegenerative disease process in MS (12). Other specific clinical aspects could also be included in the model, such as fatigue scores, cognitive tests or even non-conventional MRI biomarkers such as iron accumulation (21, 74, 75). Given the cross-sectional nature of our data, here we included gray matter (GM) volume (as opposed to atrophy) as a covariate in the statistical comparison of FC maps between groups.

More specifically, to better understand the nature of increased/decreased FC findings in MS in the present paper we investigated areas that are functionally altered and modulated by the overall lesion load (T2LL), known to be predictive of long-term disability (76). Moreover, within these areas, we questioned the relevance of compensatory mechanisms through further associations with disease severity using MSSS. In the first instance, this specific correlation study has been carried out considering the RRMS group as a whole, independently of disease duration. Results of this exploratory approach suggest indeed that the interpretation of FC decreases or increases as result of either neural disruption or compensatory brain plasticity may be an oversimplification as the scenarios presented by the data are indeed several. Associations with clinical scores of disease severity, as represented by the MSSS have been used here to help identifying possible hypothesis of FC changes in MS that we have summarized in Table 2. We suggest that areas with reduced FC and greater T2LL are considered as regions of true “*FC reductions driven* by MS *lesions”*, while network areas with increased FC but lower T2LL are considered as regions of “*true functional compensation”*. The MSSS was not investigated in these areas because it would have not changed our proposed interpretation of mechanism, based mainly on the predictive value of T2LL for long-term disability. There are counterintuitive scenarios, though, where the increased FC correlates with higher T2LL and others where a reduced FC is associated with a lower T2LL. These correlations are difficult to interpret; therefore, we propose that the sign of the correlation between FC and MSSS scores can discriminate whether FC alterations (both increased or decreased), also positively correlated with T2LL, are compensatory or maladaptive. We propose to consider as a “*functional compensation attempt”* the mechanism driving areas where an increased FC is associated with a greater T2LL in patients with a low MSSS. In other words, despite the greater damage represented by a greater T2LL, patients presenting areas satisfying this scenario are actually doing well in terms of their MSSS. When areas of increased FC and greater T2LL correlate with greater MSSS, instead, we argue that this can be considered as an indication of “*false functional compensation”* because the increased FC is associated with worse focal pathology (T2LL) and a worse clinical score (MSSS). Areas showing a decreased FC, associated with both a lower T2LL and MSSS, can be considered as areas where the functional damage may result from a “*pre-symptomatic condition”*, i.e., the functional damage (reduced FC) may precede the clinical manifestation of MS (low MSSS) and is not driven by focal damage (low T2LL). In this context, whether this scenario of reduced FC can be considered a compensatory attempt is debatable, but plausible. An interesting argument could be to interpret these changes in terms of a reduced brain functional reserve (18). On the contrary, a combination of reduced FC and T2LL associated with greater MSSS could be considered as evidence of damage due to *neurodegeneration*, i.e., the reduced FC is not caused by MS lesions (low T2LL), but may result from the presence of a coexistent non-focal neurodegenerative alteration resulting in higher clinical impairment as shown by the MSSS. Advanced microstructural and metabolic imaging, together with a longitudinal study design, could add value to the proposed mechanistic interpretation and demonstrate its validity.

Searching for areas satisfying the proposed scenarios, our data shows that only four combinations are present in this cohort (see Figure 2 and also Table S1 reported as Supplementary Material):

- *FC reductions driven* by MS *lesions*: in the inferior temporal gyrus and in the cerebellum;
- *pre-symptomatic condition*: in the frontal lobe (BA6 and BA9);
- *true functional compensation*: in the cuneus, precuneus and in the superior frontal gyrus;
- *false functional compensation*: in the cuneus and in the middle and superior frontal gyrus (in particular BA10-11).

Results do not show areas satisfying the condition of *neurodegeneration* nor the condition of *functional compensation attempt*.

**Figure 4:**
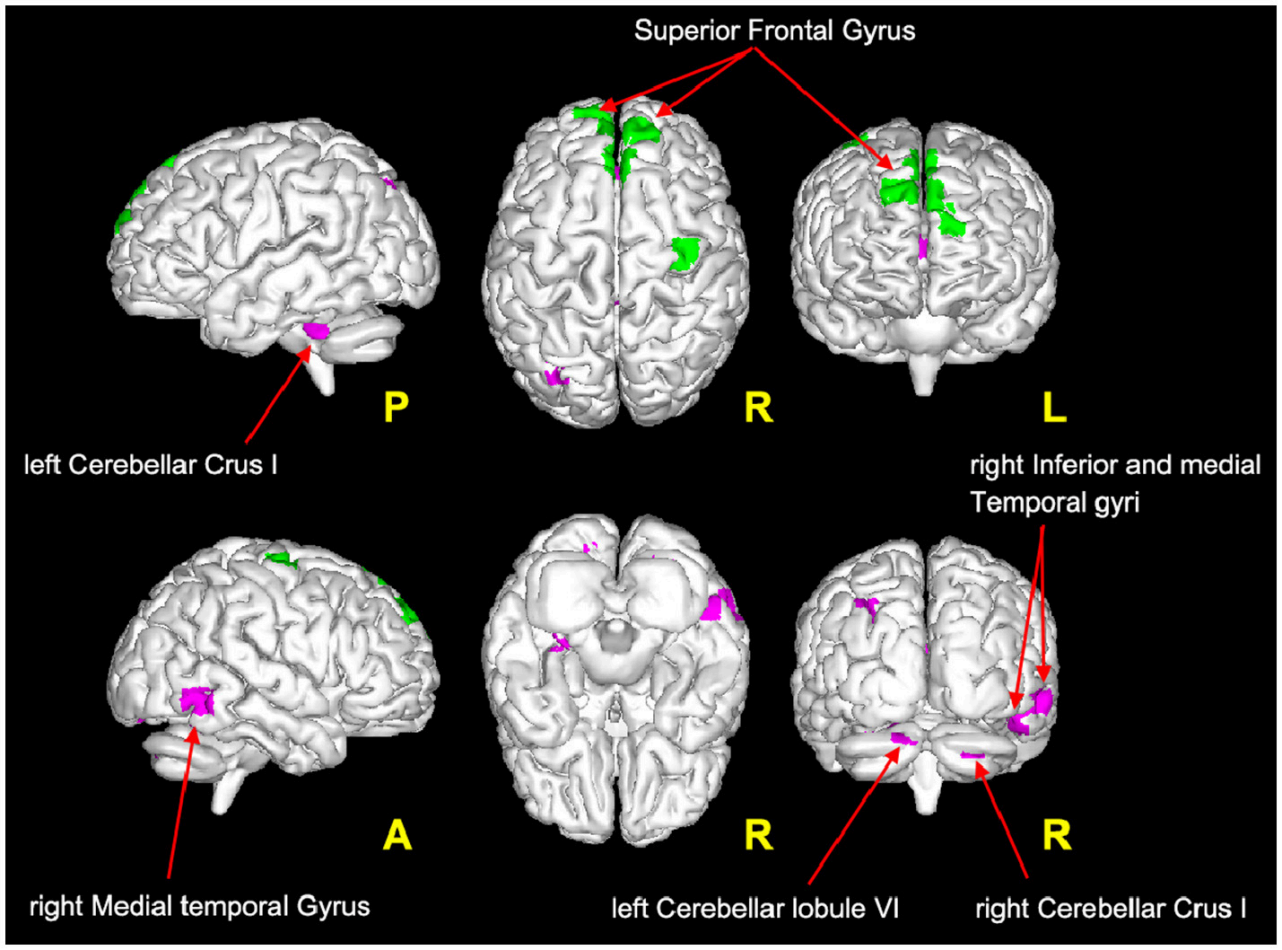
Magenta voxels: areas satisfying the condition of reduced FC driven by lesion (Table 2: *Scenario 1*), mainly located in the cerebellum (crus I and lobule VI) and in the temporal areas (middle and inferior temporal gyri). Green voxels: areas satisfying the criteria for the *pre-symptomatic condition* (Table 2: *Scenario 4b*), mainly located in the frontal lobe (superior frontal gyrus).

It is very interesting to note that according to the proposed interpretation of FC changes, areas of reduced FC in the cerebellum and in the temporal lobe satisfy the hypothesis of scenario 1 and may reflect *FC reductions driven* by MS *lesions*, while decreased FC in the frontal areas (see Figure 4) satisfies the criteria for scenario 4b and may reflect the presence of a pre-symptomatic pathological condition (i.e., functional damage prior to clinical manifestation of MS, i.e., T2LL and MSSS). Given the connectivity between the cerebellum and the frontal lobe (77) and under the assumption of the validity of the proposed multiple-scenario scheme (Table 2), one may wonder whether this pre-symptomatic reduced FC may be driven by cerebellar alterations or by thalamic atrophy, both known to be relevant in MS (12, 78, 75, 79). Future longitudinal studies may be able to answer this intriguing question. Interestingly, areas of increased FC can be found in the cuneus, precuneus, and superior frontal gyrus. Part of these areas satisfy the criteria for scenario 2, which is associated to the proposed hypothesis of true compensatory mechanism, while another non-overlapping part of them satisfy the criteria for scenario 3b, which is associated to the proposed hypothesis of false compensation. Unfortunately, the present cross-sectional study cannot inform as to the evolution of such alterations and thereby determine the consequential nature of the findings (e.g., did false compensation areas previously respond as true compensation?) or whether these are independent mechanisms of action of the pathology. However, these findings support the suggestion that increased FC has a complex nature that may exceed the simplistic assumption of an underlying compensatory mechanism attempting to limit the brain damage caused by MS evolution, exploiting or exhausting the brain functional reserve. Indeed, these considerations suggest that we cannot exclude an increased FC in MS may even represent a maladaptive response to the brain functional-structural deterioration. Another aspect to consider for future studies is the heterogeneity of the underlying T2 lesion pathology. This aspect of lesions is currently under investigation in several studies (80–82), using more specific sequences since these biophysical differences cannot be fully characterized by means of standard clinical MR scans. Dedicated sequences would help characterizing not only persistent black holes, but also different aspects of demyelination, inflammation, and microstructure alterations of lesions and to correlate them with altered FC.

Given that this was only a cross-sectional study, to investigate the possible presence of FC evolution patterns, we assessed the effect of disease duration on the FC alterations in the same mild RRMS cohort. Moreover, we believe that disease duration is often overseen to give more attention to other aspects of MS such as disability, but from the results of this study it is clear that the length of the disease affects patterns of functional alterations. In turns, understanding the mechanisms of these patterns could help understanding disease evolution. Dividing the MS cohort by disease duration provided an opportunity to study and compare rs-fMRI patterns in two clinical subgroups: (i) a subgroup in the early stage of relapsing remitting MS (disease duration < 5 years, MS_short_) and (ii) a subgroup at a later stage who have a mild relapsing remitting form of MS (disease duration >5 years, MS_long_).

Our results show that the widespread FC alterations within the RSNs differentially characterized RRMS patients depending on their disease duration. More importantly, this RRMS subjects with shorter (MS_short_) and longer (MS_long_) disease duration are differentially characterized by patterns of FC alterations affecting different large-scale networks. Specifically, results show that MS_short_ present reduced FC compared to MS_long_ subjects in a large portion of the fronto-temporo-parietal cortex involving prevalently cognitive RSNs (in particular PNsup, LNp and ECN, see Figure 3). On the other hand, MS_short_ subjects show greater FC compared to MS_long_ in more sensory areas (primary somatosensory of TPN and part of the visual areas of LVN) mainly located in the parieto-occipital cortex (Figure 3). Notably, the involvement of both sensory and cognitive RSNs in the two groups appear almost complementary (i.e., the top-ranked altered RSNs in one contrast—e.g., MS_short_ > MS_long_ – appear as the bottom-ranked altered networks in the opposite contrast—e.g., MS_short_ < MS_long_), suggesting that specific temporal dynamics may characterize MS evolution involving neuroplasticity processes and mechanisms exploiting the brain functional reserve (83). Furthermore, the areas of greater FC in MS_short_ subjects (compared to MS_long_) show the largest overlap with the map of true functional compensation identified according to the criteria of the multiple-scenario hypothesis (see Table 2). Noteworthy is that the long-term course of relapse-onset MS is variable, and in MS_short_, it is reasonable to anticipate that some will have a favorable evolution (i.e., becoming like MS_long_ subjects) while others will accumulate disability from future relapses or the development of secondary progression. However, whether marked early FC abnormalities are able to predict a less favorable disease progression is unclear, and can only be addressed in a prospective longitudinal study.

Some considerations need to be addressed with respect to the study's limitations. From a technical point of view, in order to reduce structured noise artifacts arising from head motion and physiological processes, rs-fMRI data used in this study were treated with the ICA-FIX algorithm which operates a robust but non-aggressive denoising of resting state signals (52). A further possible limitation is that the acquisition of B0-fieldmaps was not included in the MRI protocol for this study. Therefore, rs-fMRI images have not been corrected for B0-inhomogeneities during the pre-processing step. Moreover, in this study the MS group was found significantly older than the HC group (see Table 1), although the distributions of age in the two groups were strongly overlapping (see Figure S3 in Supplementary Material). Given this difference, age was added as additional covariate in the GLM model (see Materials and Methods at section Between Group RSNs Comparison and Global Alterations Ranking). Furthermore, to exclude whether the observed group differences in FC is due to age we run a further dual regression analysis using age as explanatory variable of interest, which means that the contrast vector is non-null (i.e., 1/−1 to test for positive/negative effect of age on FC) for the age column and null elsewhere in the GLM design matrix. This verification analysis resulted in no voxels surviving the significant threshold of *p* = 0.05 FWE-corrected, indicating that age does not affect the FC results we obtained in this study. Moreover, in this study significant differences were found in BDI and also in MSFC when comparing MS to HC. Interestingly, no significant difference between groups was observed in the PASAT test, which is also the third component of MSFC. Therefore, it would be worth in future studies to investigate whether the RNS FC changes between MS to HC might be influenced by their differences in BDI and MSFC. From a study design point of view, the present work is a cross-sectional investigation and although the alterations within the RSNs indicate a dysfunction of the system, influenced by focal damage as well as by disease duration, their implication for MS prognosis will require appropriate longitudinal data. In order to test the validity of the proposed interpretation of FC changes in this mild cohort of MS subjects, future studies should learn from the results and consider not only a longitudinal design, but also a multi-modal approach. The present study also investigated mechanisms of FC changes in a mild cohort of RRMS. An interesting question would be to assess a larger cohort of patients composed of different MS phenotypes, including progressive patients, to see whether our findings could be linked to brain reserve against physical disability as suggested in Sumowski et al. (84).

## Conclusions

This exploratory study investigates for the first time a voxel-wise correlation between FC and focal damage (T2LL) followed by a further voxel-wise correlation with a clinical score (MSSS). This can be considered as a basic model on which to build further analysis, for example using longitudinal measures of local atrophy (e.g., in the thalamus) or to include specific clinical or neuropsychological scores. Furthermore, this study addresses also the impact of disease duration on FC changes. As a whole, RSN FC analysis shows that functional alterations in MS at a network level cannot be simply described in terms of compensatory mechanisms or of loss of function. The analysis of FC changes in relation to overall T2 lesion load and MSSS suggests that the interpretation of FC alterations within RSNs is complex, and may include mechanisms, which involve but are not limited to true functional compensations. Of particular interest are the predominant correlations of FC reductions and T2LL in the cerebellum and the finding satisfying the proposed hypothesis of pre-symptomatic alterations in the frontal lobe, both worth further investigations. Our findings show also that FC alterations in MS are influenced by disease duration. Indeed, RRMS with shorter and longer disease duration are characterized by distinct patterns of FC alterations with a differential involvement of sensory and cognitive RSNs. Despite the limitations of a cross-sectional design, this study suggests that novel approaches to study FC alterations in multiple large-scale networks may provide new insights in the pathophysiology that underlies the evolution of relapse onset MS. Further longitudinal studies are needed to confirm our hypothesis of the mechanisms that drive FC changes in RRMS and to assess whether FC findings are able to predict the future course of the disease.

## Author contributions

GC, LD, CW-K, and DFM conceptualized the study. GC designed and performed the analyses with support LD. TM and DFM acquired all MRI data. LD, JD-A, and DFM enrolled patients and acquired all the clinical and neuro-radiological data helping for data interpretation. DHM, CW-K, ED, JD-A, and DFM provided support and guidance with data interpretation with the clinical contribution of all physicians. GC, CW-K, and DHM wrote the manuscript, with comments from all other authors.

### Conflict of interest statement

DHM received honoraria through payments to his employer, UCL Institute of Neurology, for Advisory Committee and/or Consultancy advice in multiple sclerosis studies from Biogen Idec, Novartis, Mitsubishi Pharma Europe and Bayer Schering Pharma. DHM also received compensation through payments to his employer for performing central MRI analysis of multiple sclerosis trials from Biogen Idec, Novartis and Apitope. CW-K received research funding from EPSRC, UK MS Society, Horizon2020, Biogen Idec, Novartis, Wings for Life, Spinal Research and CHNF. DFM has received honoraria and travel grants from Biogen Idec, Novartis and TEVA.

The remaining authors declare that the research was conducted in the absence of any commercial or financial relationships that could be construed as a potential conflict of interest.
