# Supplementary material for: Functional Connectivity Alterations Reveal Complex Mechanisms Based on Clinical and Radiological Status in Mild Relapsing Remitting Multiple Sclerosis
Source: Front Neurol. 2018 Aug 20;9:690. doi: 10.3389/fneur.2018.00690 (PMC6109785; doi:10.3389/fneur.2018.00690)
Supplement: Supplementary file 1 [file Presentation_1.PDF]

frontiers

Supplementary Material

Functional connectivity changes reveal complex mechanisms based on clinical and radiological status in mild relapsing remitting multiple sclerosis

G. Castellazzi*, L. Debernard, T.R. Melzer, J.C. Dalrymple-Alford, E. D'Angelo, D.H. Miller, C.A.M. Gandini Wheeler-Kingshott, D.F. Mason
*** Correspondence:** Gloria Castellazzi: gloria.castellazzi@unipv.it

1 Supplementary Figures and Tables

1.1 Supplementary Figures

Figure S1: Flowchart of the correlation analyses performed between altered FC, T2LL and MSSS. The diagram has the purpose to help reconstructing a possible model of FC changes interpretation.

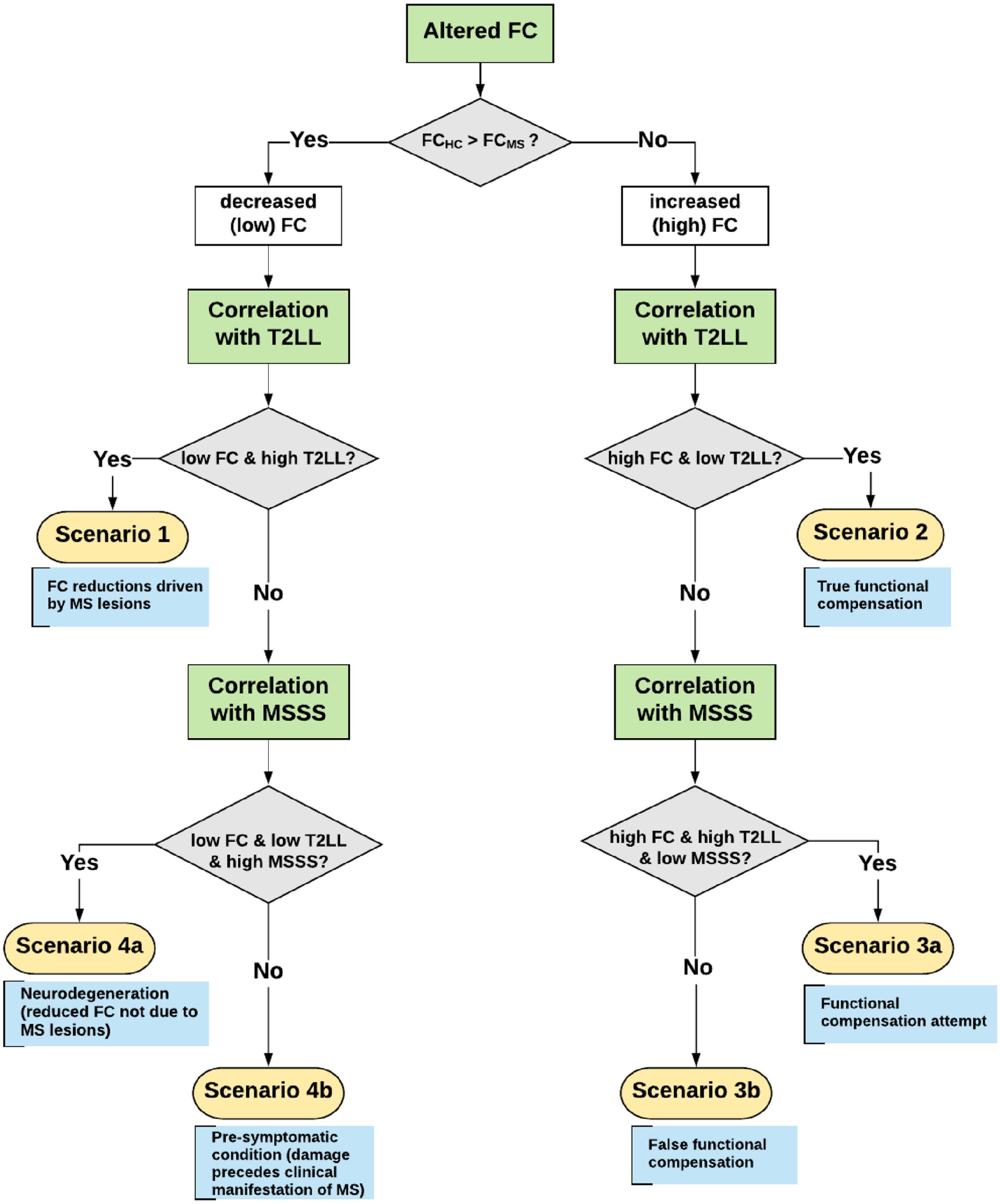
Altered FC
FC_HC > FC_MS ?
Yes
No
decreased (low) FC
increased (high) FC
Correlation with T2LL
Correlation with T2LL
low FC & high T2LL?
high FC & low T2LL?
Yes
Yes
Scenario 1
FC reductions driven by MS lesions
Scenario 2
True functional compensation
No
No
Correlation with MSSS
Correlation with MSSS
low FC & low T2LL & high MSSS?
high FC & high T2LL & low MSSS?
Yes
Yes
Scenario 4a
Neurodegeneration (reduced FC not due to MS lesions)
Scenario 3a
Functional compensation attempt
No
No
Scenario 4b
Pre-symptomatic condition (damage precedes clinical manifestation of MS)
Scenario 3b
False functional compensation

Figure S2: Maps of the 18 RSNs identified from ICA outputs. Each network is projected onto three representative sections chosen on the sagittal (left), coronal (middle) and axial (right) planes.

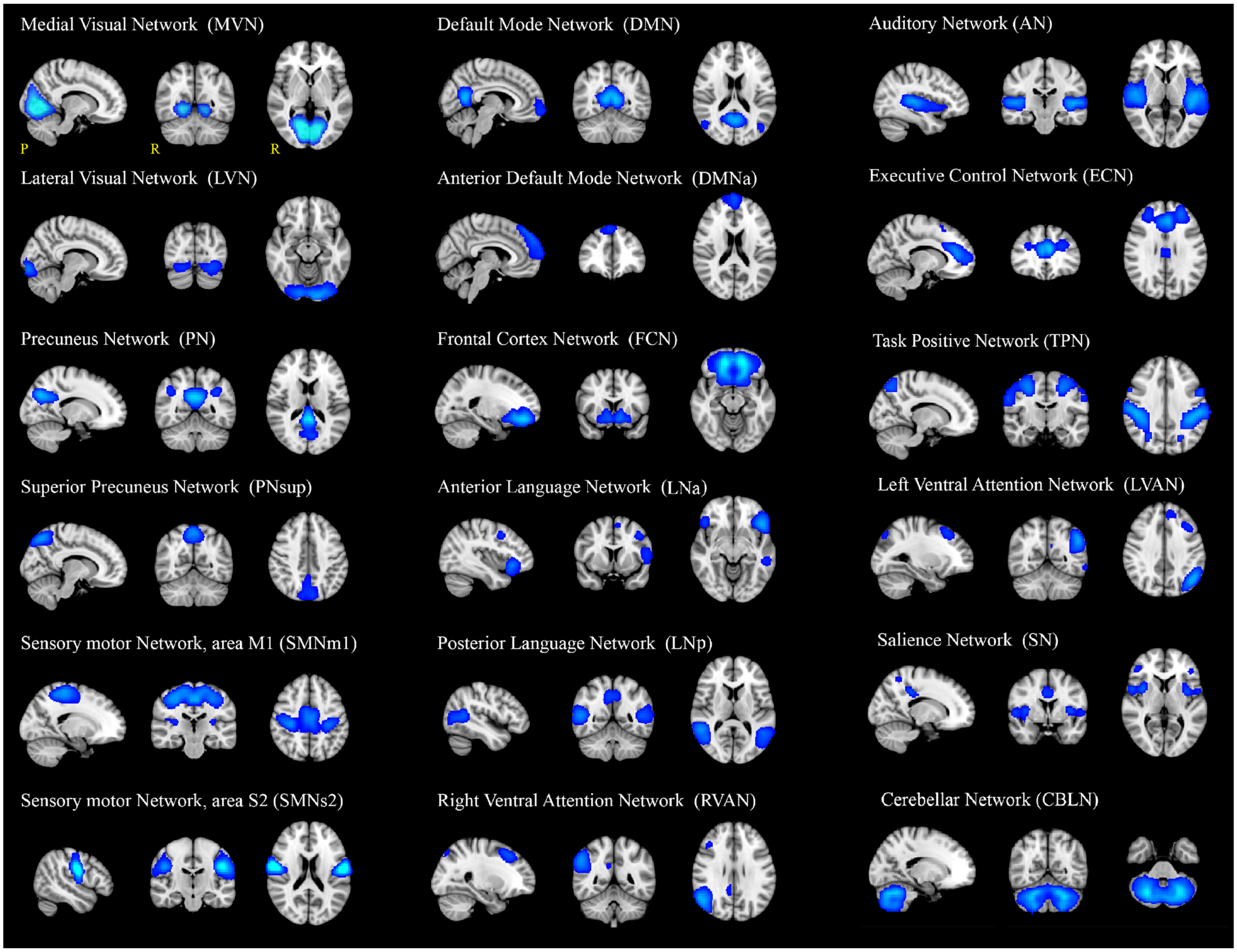

Figure S3: **(a,b)** age distributions (histograms and relative normal curves) of age in the HC group (b) and in the MS group (b). **(c)** Overlapping between the age distributions of HC (in blue) and MS (in red).

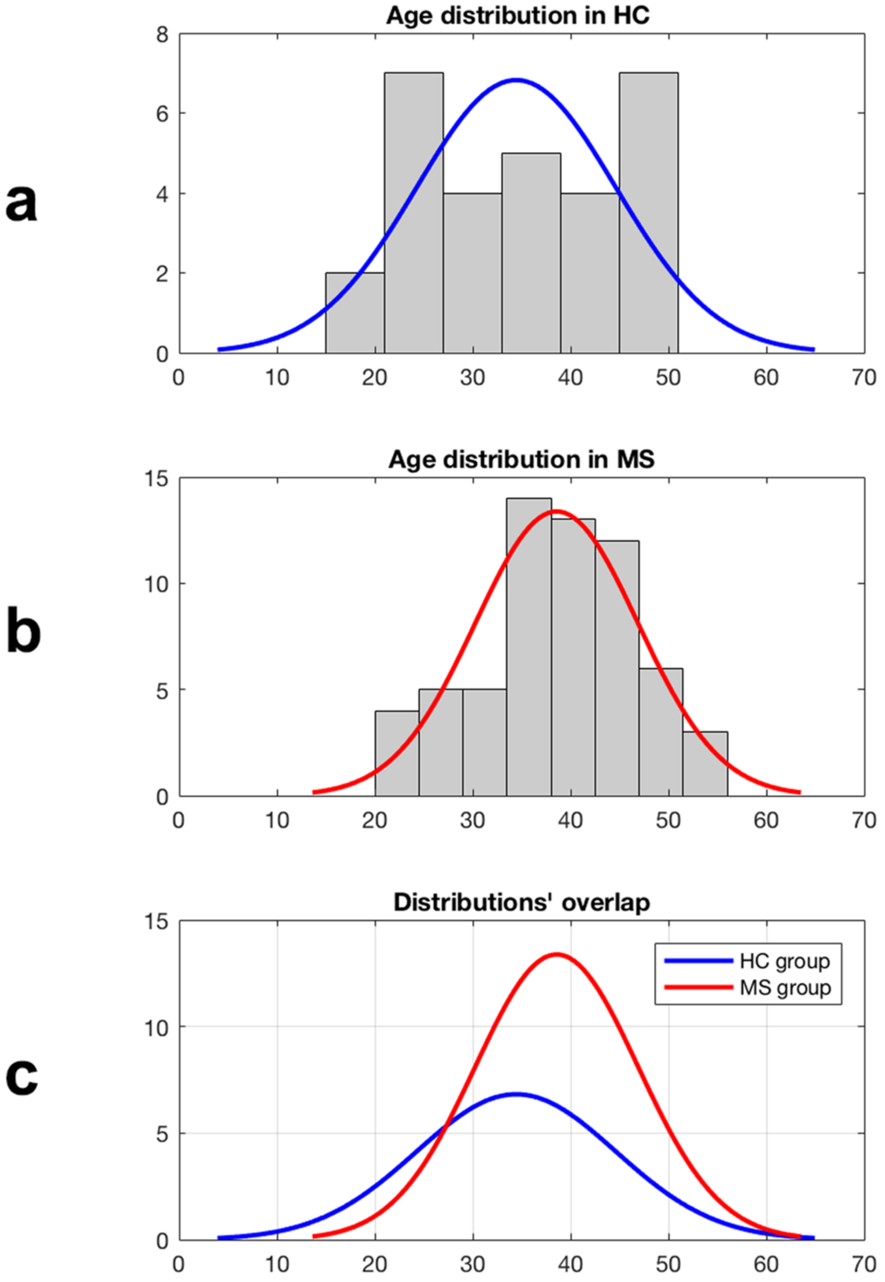

1.2 Supplementary Tables

Table S1: Detail of the anatomical location of the FC changes within the RSNs in MS compared to HC interpreted according to the multiple scenario proposed in Table 2.

Mechanism	**FC changes in RSNs (clusters' centroids)**
FC reductions driven by MS lesions	**MVN** (left posterior cerebellar declive)<br>**LVN** (BA37, right inferior and middle temporal gyrus, fusiform gyrus)<br>**SN** (left cerebellar crus I, posterior cerebellum)<br>**TPN** (left cerebellar crus I, posterior cerebellum)<br>**PN** (right posterior cingulate cortex, BA19, left inferior and middle occipital gyrus)<br>**CBLN** (left cerebellar lobule VI, anterior cerebellum)
True functional compensation	**MVN** (left calcarine, BA30, cuneus)<br>**LVN** (right superior occipital gyrus, cuneus, BA18, BA19)<br>**LVAN** (left angular gyrus, inferior parietal lobule)<br>**ECN** (left superior and medial frontal gyrus)<br>**SMNm1** (left precuneus, BA7, secondary sensorimotor cortex)
Functional compensation attempt	*No evidence*
False functional compensation	**MVN** (left superior occipital gyrus, BA19, cuneus)<br>**ECN** (left anterior cingulate cortex, BA10, BA32, middle frontal gyrus)<br>**LNa** (left inferior frontal gyrus, pars opercularis, BA44, precentral gyrus)<br>**FCN** (right superior and middle frontal gyrus, pars orbitalis, BA11)
Neurodegeneration (reduced FC not due to MS lesions)	*No evidence*
Pre-symptomatic condition (signal of damage is previous to clinical manifestation of MS)	**DMNa** (right medial and superior frontal gyrus, BA9)<br>**TPN** (right precentral gyrus, BA6)
